# Prognostic implications of prostaglandin E-major urinary metabolite in resected non-small-cell lung cancer

**DOI:** 10.1093/icvts/ivac291

**Published:** 2023-01-09

**Authors:** Masashi Mikubo, Yukitoshi Satoh, Mototsugu Ono, Dai Sonoda, Shoko Hayashi, Masahito Naito, Yoshio Matsui, Kazu Shiomi, Masaaki Matsuura, Satoru Ito

**Affiliations:** Department of Thoracic Surgery, Kitasato University School of Medicine, Sagamihara, Japan; Department of Thoracic Surgery, Kitasato University School of Medicine, Sagamihara, Japan; Department of Thoracic Surgery, Kitasato University School of Medicine, Sagamihara, Japan; Department of Thoracic Surgery, Kitasato University School of Medicine, Sagamihara, Japan; Department of Thoracic Surgery, Kitasato University School of Medicine, Sagamihara, Japan; Department of Thoracic Surgery, Kitasato University School of Medicine, Sagamihara, Japan; Department of Thoracic Surgery, Kitasato University School of Medicine, Sagamihara, Japan; Department of Thoracic Surgery, Kitasato University School of Medicine, Sagamihara, Japan; Graduate School of Public Health, Teikyo University, Tokyo, Japan; IDAC Theranostics, Inc, Tokyo, Japan

**Keywords:** Prostaglandin E-major urinary metabolite, Non-small-cell lung cancer, Surgery, Biomarker, Adjuvant chemotherapy

## Abstract

**OBJECTIVES:**

Cyclooxygenase-2-derived prostaglandin E2 (PGE2) is highly involved in the promotion of cancer progression. The end product of this pathway, PGE-major urinary metabolite (PGE-MUM), is a stable metabolite of PGE2 that can be assessed non-invasively and repeatedly in urine samples. The aim of this study was to assess the dynamic changes in perioperative PGE-MUM levels and their prognostic significance in non-small-cell lung cancer (NSCLC).

**METHODS:**

Between December 2012 and March 2017, 211 patients who underwent complete resection for NSCLC were analysed prospectively. PGE-MUM levels in 2 spot urine samples taken 1 or 2 days preoperatively and 3–6 weeks postoperatively were measured using a radioimmunoassay kit.

**RESULTS:**

Elevated preoperative PGE-MUM levels were associated with tumour size, pleural invasion and advanced stage. Multivariable analysis revealed that age, pleural invasion, lymph node metastasis and postoperative PGE-MUM levels were independent prognostic factors. In matched pre- and postoperative urine samples obtained from patients who are eligible for adjuvant chemotherapy, an increase in PGE-MUM levels following resection was an independent prognostic factor (hazard ratio 3.017, *P* = 0.005). Adjuvant chemotherapy improved survival in patients with increased PGE-MUM levels after resection (5-year overall survival, 79.0 vs 50.4%, *P* = 0.027), whereas survival benefit was not observed in those with decreased PGE-MUM levels (5-year overall survival, 82.1 vs 82.3%, *P* = 0.442).

**CONCLUSIONS:**

Increased preoperative PGE-MUM levels can reflect tumour progression and postoperative PGE-MUM levels are a promising biomarker for survival after complete resection in patients with NSCLC. Perioperative changes in PGE-MUM levels may aid in determining the optimal eligibility for adjuvant chemotherapy.

**Figure:**
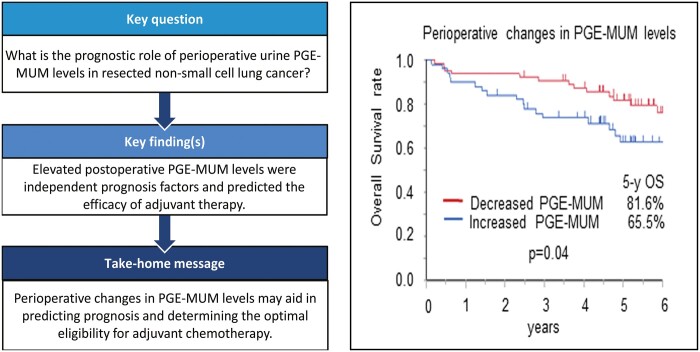

## INTRODUCTION

Lung cancer is the leading cause of cancer-related death worldwide. Although active and passive smoking are the primary risk factors for lung cancer, several occupational and environmental factors and pre-existing lung diseases such as chronic obstructive pulmonary disease (COPD) and idiopathic pulmonary fibrosis are also involved [1]. Under these conditions, many environmental factors and cancer risk factors are associated with chronic inflammation. Inflammatory responses play integral roles at different stages of tumour development, including initiation, promotion, invasion and metastasis and also affect immune surveillance and responses to therapy [2].

In inflammatory and tumourigenic signalling, prostaglandin E2 (PGE2) is synthesized from arachidonic acid via a cyclooxygenase (COX)-catalyzed reaction [3]. Since the lungs play an important role in both the production and metabolism of PGE2, PGE2 levels in clinical specimens are possible indicators of inflammation-associated lung disorders [4]. Furthermore, COX-2-derived PGE2 is reportedly the most significant prostaglandin involved in cancer progression [3, 5]. Although previous studies investigating the association between COX-2/PGE2 activity in tumour tissue and tumour progression have been conducted, accurately measuring its expression in tissue samples has several limitations, such as its rapid degradation, invasiveness of sampling and tumour heterogeneity [6, 7].

Prostaglandin E-major urinary metabolite (PGE-MUM) is a stable end product of PGE2 metabolism and can be measured in urine samples [8]. Its measurement allows for non-invasive longitudinal evaluation and can reflect systemic COX-2/PGE2 activity. A recent study demonstrated that PGE-MUM levels were significantly elevated in patients with lung adenocarcinoma and tended to be positively correlated with cancer progression [9]. However, dynamic changes in PGE-MUM during the perioperative period have not been evaluated to date, and their prognostic significance remains unclear. In the present study, we prospectively investigated the prognostic impact of perioperative PGE-MUM levels in patients with resected non-small-cell lung cancer (NSCLC) and discussed the significance thereof as a clinical biomarker.

## PATIENTS AND METHODS

### Ethical statement

The ethics committees of Kitasato University (B12-119) and Teikyo University (Teirin-19-243) approved this study, which was conducted in accordance with the Declaration of Helsinki. Informed consent was obtained from all participants.

### Collection of samples and data

In total, 373 patients who underwent complete resection for primary NSCLC at Kitasato University Hospital, Japan, between December 2012 and March 2017 were prospectively enrolled in the present study. Patients with carcinoma in situ, non-curative resection, synchronous or metachronous (within 5 years) malignancies, clinically or pathologically confirmed distant metastasis or pleural dissemination were excluded. Comorbidities were scored using Charlson comorbidity index [10]. All resected tumours were histologically diagnosed using the WHO classification and staged in accordance with the seventh edition of the TNM classification staging manual [11, 12]. Adjuvant chemotherapy was considered for patients with pathologic stage IA (tumour size >2 cm), IB–IIIA NSCLC according to the Japanese guideline [13].

Two spot urine samples were obtained from patients with primary lung cancer in the morning at 1 or 2 days preoperatively and 3–6 weeks postoperatively. In patients who received adjuvant chemotherapy, postoperative urine samples were obtained before the initiation of chemotherapy. All samples were centrifuged at 1000*g* for 10 min, and the supernatants were stored at −20°C until analysis.

The prognostic impact of absolute pre- and postoperative PGE-MUM levels and perioperative changes of PGE-MUM levels were investigated separately. Therefore, patients whose only preoperative urine samples were available for PGE-MUM analysis were included in the present study. Among all cases, only patients whose matched pre- and postoperative PGE-MUM levels were available were included in the analysis of perioperative changes in PGE-MUM levels.

### Measurement of urinary prostaglandin E-major urinary metabolite levels

Each urine sample was tested individually using the PGE-MUM assay. A radioimmunoassay kit (Institute of Isotopes Co., Ltd, Budapest, Hungary) was used to measure the PGE-MUM levels. Briefly, the synthesized level of bicyclic PGE-MUM was measured using a competitive assay after alkaline treatment was performed, followed by neutralization of the urine samples. PGE-MUM levels were normalized to the concentration of urinary creatinine (expressed as μg/g Cr) because PGE-MUM concentration depends on urinary volume.

### Statistical analysis

Pre- and postoperative PGE-MUM levels were compared based on clinicopathological characteristics. All continuous variables identified with abnormal distribution by the Shapiro–Wilk test were analysed with Mann–Whitney *U*-test. Multivariable Cox regression models were used to examine prognostic factors. Survival was calculated using the Kaplan–Meier method, and differences were determined by means of log-rank analysis. The variables with a univariable *P*-value of <0.20 were included in the multivariable model. Multicollinearity between variables in the multivariable model was assessed using a variance inflation factor. The goodness of fit of the models was evaluated using the Akaike information criterion (AIC) and the Bayesian information criterion (BIC). The AIC and BIC are model selection criteria and calculated as follows: AIC = −2 log (*L*) + 2*k*, BIC = −2 log (*L*) + *k* log (*n*). In this formula, *n* is the number of samples, *L* is the likelihood for the mode and *k* is the number of parameters in the model [14]. When variables are selected in multivariable analysis, the number of samples changes owing to missing values. Since the normally used AIC can only be used when the number of samples is the same, we statistically selected a model based on the BIC that can be compared even if the number of samples is different. Missing data ware handled by listwise deletion. A *P*-value of <0.05 was considered to indicate statistical significance. Statistical analyses were performed using JMP Pro version 15.0 (SAS Institute, Cary, NC, USA).

## RESULTS

### Patient characteristics

The clinicopathological data of 369 consecutive patients with NSCLC included in the present study were retrieved from medical records. Among them, 157 patients who were treated with laxatives were excluded because laxative administration induces PGE2 production [15]. Accordingly, the relevant spot urine PGE-MUM levels were measured in the remaining 211 patients with NSCLC (130 men and 81 women). The clinicopathological characteristics of these 211 patients are presented in Table 1. None of the patients presented with inflammatory bowel diseases, acute cardiovascular problems and severe renal dysfunction in which urine PGE-MUM levels were positively affected. Of the 211 patients, 90 (43%) and 15 (7%) had COPD and idiopathic pulmonary fibrosis, respectively. A total of 178 patients (84.3%) received non-steroidal anti-inflammatory drugs (NSAIDs) during a postoperative course. Adenocarcinoma was the most common lung cancer (79%), followed by squamous cell carcinoma (13%), large-cell neuroendocrine carcinoma (4%) and others (5%). In total, 141 (67%) patients had pathologic stage I disease (91 with stage IA and 50 with stage IB), while 70 had more advanced disease (20, 24 and 26 with stages IIA, IIB and IIIA, respectively). Adjuvant chemotherapy was administered to 66 (31%) patients. Chemotherapy regimens included platinum doublet in 41 patients and tegafur-uracil in 25.

**Table 1:** Clinicopathologic characteristics of patients with non-small-cell lung cancer (*N* = 211)

Variables	Value, *n* (%)
Age (years) 67.4 [SD]	67.5 (9.7)
Sex	
Male	130 (62)
Female	81 (38)
Smoking history	
Current or former	134 (64)
Never	77 (36)
Smoking index, mean	541.4
NSAIDs administration	
Yes	178 (84)
No	33 (16)
Charlson comorbidity index	
0	117 (55)
1	68 (32)
≥2	26 (12)
COPD	
Yes	90 (43)
No	121 (57)
IPF	
Yes	15 (7)
No	196 (93)
Tumour size, mean (SD)	33.3 (22.5)
Histology	
Adenocarcinoma	166 (79)
Squamous cell carcinoma	27 (13)
Large cell neuroendocrine carcinoma	8 (4)
Others	10 (5)
Pleural invasion	
Yes	66 (31)
No	145 (69)
Pathologic N status	
N0	172 (82)
N1	15 (7)
N2	24 (11)
Pathologic stage	
IA	91 (43)
IB	50 (24)
IIA	20 (10)
IIB	24 (11)
IIIA	26 (12)
Adjuvant chemotherapy	
Yes	66 (31)
No	145 (69)

COPD: chronic obstructive pulmonary disease; IPF: idiopathic pulmonary fibrosis; NSAIDs: non-steroidal anti-inflammatory drugs; SD: standard deviation.

### Prostaglandin E-major urinary metabolite levels in pre- and postoperative urinary samples

PGE-MUM levels were evaluated using spot urine samples obtained pre-(*n* = 211) and postoperatively (*n* = 196). Fifteen patients had only preoperative urine samples available for PGE-MUM analysis. The average PGE-MUM levels were 19.8 and 17.9 µg/g Cr in pre- and postoperative samples, respectively. These levels were elevated compared with those in healthy volunteers (15.4 µg/g Cr) [9]. The pre- and postoperative PGE-MUM levels in the clinical samples are summarized in Table 2. Preoperative PGE-MUM levels were associated with sex, smoking history and the presence of COPD but not histological cancer type, NSAIDs administration and comorbidity score. Patients with large tumours (≥33 mm, *P* = 0.002), tumour with pleural invasion (*P* = 0.0002) and advanced-stage disease (*P* = 0.0006) exhibited significantly increased preoperative PGE-MUM levels, suggesting that PGE-MUM before tumour resection reflects tumour burden and progression. However, absolute postoperative PGE-MUM levels were not associated with any clinical or pathological characteristics including NSAIDs administration.

**Table 2:** Pre- and postoperative prostaglandin E-major urinary metabolite levels in patients with non-small-cell lung cancer

Variables	Before surgery			After surgery		
	*N*	Average PGE-MUM (µg/g Cr)	*P*-Value	*N*	Average PGE-MUM (µg/g Cr)	*P*-Value
Sex						
Male	130	22.2	0.006	120	18.2	0.62
Female	81	15.9		76	17.4	
Smoking history						
Current or former	134	22.4	0.002	124	17.9	0.95
Never	77	15.3		72	18.0	
NSAIDs administration						
Yes	178	19.5	0.55	167	18.1	0.65
No	33	21.3		29	17.0	
Charlson comorbidity index						
0	117	20.7	0.57	112	18.0	0.52
1	68	18.1		59	16.9	
≥2	26	19.8		25	19.7	
COPD						
Yes	90	23.1	0.01	83	19.3	0.15
No	121	17.3		113	16.9	
IPF						
Yes	15	25.9	0.13	15	23.1	0.07
No	196	19.3		181	17.5	
Histology						
Adenocarcinoma	166	19.1	0.23	155	17.9	0.94
Non-adenocarcinoma	45	22.4		41	18.0	
Tumour size						
≥33 mm	76	24.2	0.002	70	18.4	0.60
<33 mm	135	17.3		126	17.6	
Pleural invasion						
Yes	66	25.8	0.0002	61	18.3	0.75
No	145	17.0		135	17.7	
Lymph node metastasis						
Yes	39	22.1	0.33	37	16.5	0.39
No	172	19.3		159	18.2	
Pathologic stage						
Stage I	141	17.1	0.0002	131	18.3	0.78
Stage II	44	19.7		40	16.9	
Stage III	26	28.4		25	17.5	

COPD: chronic obstructive pulmonary disease; IPF: idiopathic pulmonary fibrosis; NSAIDs: non-steroidal anti-inflammatory drugs; PGE-MUM: prostaglandin E-major urinary metabolite.

### Prognostic effect of prostaglandin E-major urinary metabolite in non-small-cell lung cancer

**Figure 1:**
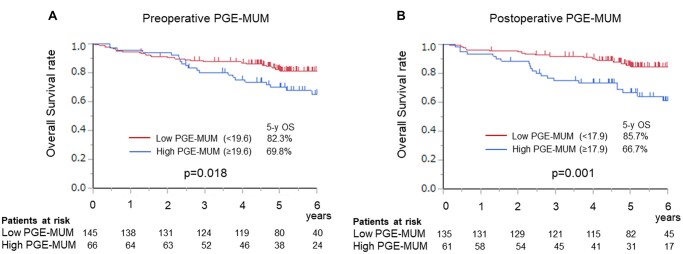
(**A**) Overall survival curve according to preoperative PGE-MUM levels. (**B**) Overall survival curve according to postoperative PGE-MUM levels. PGE-MUM levels were divided based on mean values (19.6 and 17.9 µg/g Cr in pre- and postoperative samples, respectively). PGE-MUM: prostaglandin E-major urinary metabolite

The median follow-up duration following resection was 51.4 months. A total of 37 patients experienced recurrence at distant sites, while 38 experienced loco-regional recurrence. Among the 211 patients, 47 died during the follow-up period, 26 of lung cancer and 21 of other causes. To investigate the role of PGE-MUM as a prognostic factor, we compared the prognosis of patients with high PGE-MUM levels with that of patients with low PGE-MUM levels. The levels were divided based on mean values, 19.6 µg/g Cr in preoperative samples and 17.9 µg/g Cr in postoperative samples. Patients with high PGE-MUM levels before and after resection exhibited significantly worse overall survival (OS) (5-year OS, 82.3 vs 69.8%, *P* = 0.018 and 5-year OS, 85.7 vs 66.7%, *P* = 0.001, respectively) (Fig. 1A and B). This trend was observed regardless of receipt of NSAIDs (Supplementary Material, Fig. S1) However, despite the close association with tumour progression and negative prognostic impact in the univariable analysis, preoperative PGE-MUM was not an independent prognostic factor in the multivariable analysis for OS (Table 3). Notably, postoperatively, the PGE-MUM level remained a significant prognostic factor [hazard ratio (HR), 6.462; 95% confidence interval (CI): 1.284–32.49, *P* = 0.024] as did age (HR 3.083, 95% CI: 1.076–8.836, *P* = 0.036), pleural invasion (HR 2.269, 95% CI: 1.165–4.383, *P* = 0.015) and lymph node metastasis (HR 2.216, 95% CI: 1.101–4.459, *P* = 0.026), suggesting that preoperative PGE-MUM could be a predictive marker for tumour progression at the time of surgery but does not affect prognosis independently, whereas postoperative PGE-MUM may be a prognostic marker in patients with resected NSCLC.

**Table 3:** Multivariable Cox regression analysis of factors influencing overall survival in resected non-small-cell lung cancer

Variable	Hazard ratio	95% confidence interval		*P*-Value
		Lower	Upper	
Age (years) (≥65/<65)	3.083	1.076	8.836	0.036
Sex (male/female)	1.608	0.771	3.352	0.205
Tumour size (≥33 mm/<33 mm)	1.399	0.720	2.719	0.321
Pleural invasion (present/absent)	2.269	1.165	4.383	0.015
LN metastasis (present/absent)	2.216	1.101	4.459	0.026
Preoperative PGE-MUM (µg/g Cr)	1.820	0.135	24.47	0.651
Postoperative PGE-MUM (µg/g Cr)	6.462	1.284	32.49	0.024

LN: lymph node; PGE-MUM: prostaglandin E-major urinary metabolite.

### Implications of perioperative changes in prostaglandin E-major urinary metabolite levels and indication for adjuvant chemotherapy

**Figure 2:**
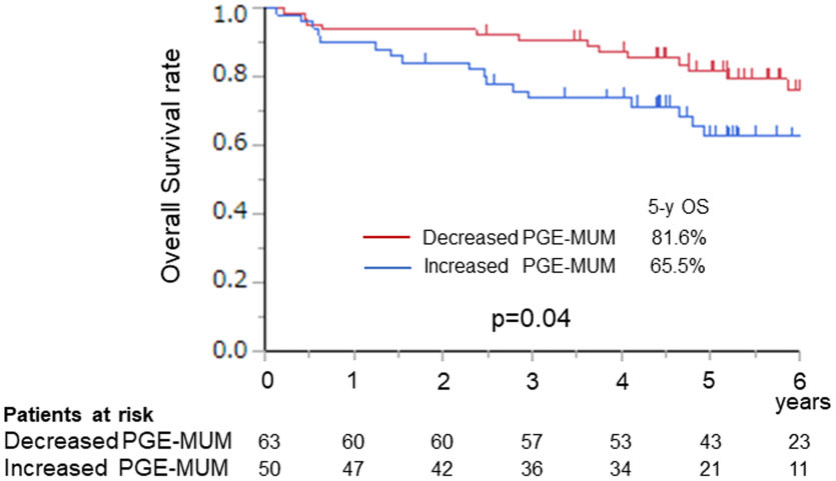
Overall survival curve according to the change in postoperative PGE-MUM levels compared with preoperative PGE-MUM levels. PGE-MUM: prostaglandin E-major urinary metabolite

**Figure 3:**
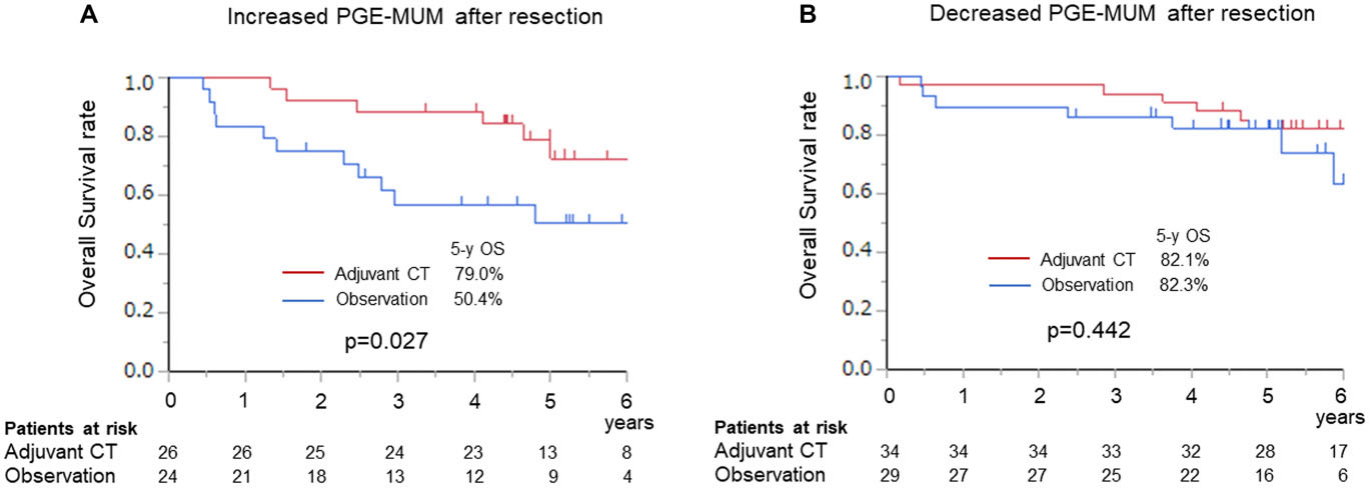
(**A**) Overall survival in patients with increased PGE-MUM levels after resection who received adjuvant chemotherapy and those who did not. (**B**) Overall survival in patients with decreased PGE-MUM levels after resection who received adjuvant chemotherapy and those who did not. PGE-MUM: prostaglandin E-major urinary metabolite.

Since high PGE-MUM levels after resection were associated with poor prognosis in patients with NSCLC, we investigated the association between pre- and postoperative PGE-MUM changes and prognosis and assessed possible indication for adjuvant therapies based on PGE-MUM changes. Matched pre- and postoperative PGE-MUM levels were available for 113 of 127 patients who were considered eligible for adjuvant chemotherapy according to the clinical guideline. Of the 113 patients, 60 received adjuvant chemotherapy and 53 did not. Fifty patients experienced elevated PGE-MUM levels after resection relative to preoperative levels, with an average increase of 6.45 µg/g Cr. A reduction was observed in 63 patients, with an average reduction of 12.76 µg/g Cr. The OS of patients with increased PGE-MUM levels after resection was significantly worse than that of patients with decreased PGE-MUM levels (5-year OS, 81.6 vs 65.5%, *P* = 0.04) (Fig. 2). In multivariable analysis for OS, an increase in PGE-MUM levels after resection was an independent prognostic factor (HR, 2.272; 95% CI: 1.038–4.766, *P* = 0.039) along with pleural invasion, lymph node metastasis and adjuvant chemotherapy (Table 4). In patients with increased PGE-MUM levels after resection, adjuvant chemotherapy significantly improved survival (5-year OS, 79.0 vs 50.4%, *P* = 0.027) (Fig. 3A). However, in patients with decreased PGE-MUM levels after resection, there were no significant differences in OS between patients who received adjuvant chemotherapy and those who did not (5-year OS, 82.1 vs 82.3%, *P* = 0.442) (Fig. 3B).

**Table 4:** Multivariable Cox regression analysis of factors influencing overall survival in resected non-small-cell lung cancer with matched pre- and postoperative prostaglandin E-major urinary metabolite examination

Variable	Hazard ratio	95% confidence interval		*P*-Value
		Lower	Upper	
Age (years) (≥65/<65)	3.672	1.076	12.534	0.038
Sex (male/female)	1.759	0.669	4.626	0.253
Tumour size (mm) (≥33/<33)	1.605	0.738	3.489	0.232
Pleural invasion (present/absent)	3.453	1.532	7.782	0.003
LN metastasis (present/absent)	2.865	1.317	6.229	0.008
Increase of PGE-MUM after resection (yes/no)	2.272	1.038	4.766	0.039
Adjuvant therapy (no/yes)	2.400	1.041	5.533	0.040

LN: lymph node; PGE-MUM: prostaglandin E-major urinary metabolite.

## DISCUSSION

In the present study, we demonstrated that elevated preoperative PGE-MUM levels were associated with cancer advancement at the time of surgery and postoperative PGE-MUM levels were a promising biomarker for poor prognosis after complete resection in patients with NSCLC. These results may provide useful information for perioperative management including postoperative systemic therapy.

PGE-MUM is a urinary metabolite of PGE2 and represents the activity of the COX-2 pathway. COX-2 and COX-2-derived PGE2 are reportedly involved in the tumour initiation and proliferation of cancer cells both *in vitro* and *in vivo* [5, 16]. Several studies performed in clinical settings have shown that COX-2 overexpression is associated with poor prognosis in solid tumours, including NSCLC [6, 17]. Furthermore, based on the hypothesis that suppressing the COX-2 pathway could reduce cancer aggression and improve survival, prospective clinical studies have been conducted to examine the effects of COX-2 inhibitors combined with standard chemotherapies. However, no randomized controlled trial has demonstrated a therapeutic advantage for these agents [7, 18–21]. Considerable limitations discussed in these studies included optimal patient selection based on clinical biomarkers and the methods used to detect the markers. In a phase III study, CALGB 30801, the enrolled patients were selected based on COX-2 expression assessed using immunohistochemistry, and the COX-2 inhibitor celecoxib failed to improve prognosis in NSCLC with high COX-2 expression. However, a cohort of patients who could benefit from celecoxib was identified in subset analysis using a urinary PGE2 metabolite. Correlation and agreement were poor between urinary PGE2 metabolite and COX-2 staining by immunohistochemistry, and prognosis in NSCLC with elevated urinary PGE metabolite was improved by celecoxib treatment [7]. Given the advantage of urinary samples, which enable repeated real-time assessment and do not require consideration of tumour heterogeneity in biomarker expression, PGE-MUM could be promising for evaluating the activity of the COX-2 pathway and reflecting the exact biological features of tumours.

Despite evidence on the association between COX-2-derived PGE2 and prognosis in malignant tumours, the dynamics and prognostic significance of systemic COX-2 activity during the perioperative period remain unclear. The present study demonstrated that high PGE-MUM levels after resection were associated with poor prognosis in patients with NSCLC. This result could be underpinned by 2 possible implications: the cancer-promoting effect of inflammation and the presence of microscopic residual tumour cells, called minimal residual disease (MRD). Inflammation has several tumour-promoting effects, including proliferation, angiogenesis, invasiveness and metastasis [22]. The inflammatory burden in patients undergoing curative resection for lung cancer reportedly affects survival negatively [23, 24]. As postoperative PGE-MUM was an independent prognostic factor in our study regardless of tumour progression, prolonged excessive inflammation after surgery might facilitate the survival and development of microscopic tumour cells, which could subsequently lead to tumour recurrence and poor prognosis. Another interpretation is the role of PGE-MUM as a biomarker for residual tumour cells. Since preoperative PGE-MUM reflects tumour burden and high postoperative PGE-MUM could be associated with poor prognosis, the lack of reduction in PGE-MUM after surgery is assumed to represent the presence of microscopic residual tumour cells. Although inflammation can promote tumour progression, tumours can also induce inflammation by secreting inflammatory cytokines into their microenvironment to maintain favourable conditions for their growth. PGE2 can reportedly be secreted from tumour cells, thereby promoting immune evasion [25]. Thus, high postoperative PGE-MUM levels and subsequent poor prognosis may be a consequence of MRD with activation of the COX-2/PGE2 pathway.

With the recent exploration of biomarkers and development of drugs for unresectable lung cancers, greater attention has been paid to perioperative treatment tailored based on biomarkers. Although indications for adjuvant therapies are primarily determined based on pathological stage, even tumours with the same advanced stage and histologic type represent a highly heterogeneous population. This includes patients who do not truly require adjuvant therapy. Recent studies attempting to identify tumours with a high risk for postoperative recurrence and select optimal indications for adjuvant therapies using developed detection methods for MRD after surgical resection have been conducted [26–28]. Based on the presumption that sustained elevated PGE-MUM levels after resection represent the presence of MRD, we investigated the association between pre- and postoperative PGE-MUM changes and prognosis to assess possible indication for adjuvant therapies based on PGE-MUM levels. Multivariable analysis revealed that an increase in PGE-MUM levels after resection remained a significant prognostic factor. Furthermore, adjuvant chemotherapy improved OS in patients with increased PGE-MUM levels after resection, while the survival benefit from adjuvant chemotherapy was not observed in patients with decreased PGE-MUM levels after resection. These results may support a role of perioperative changes in PGE-MUM levels as a clinical biomarker representing the presence of MRD and provide useful information for determining the optimal eligibility for adjuvant therapy to improve the survival of patients with NSCLC.

### Limitations

The present study had several limitations. First, the investigation was conducted at a single centre with a limited number of enrolled patients. Second, PGE-MUM levels may be affected by several inflammatory conditions. Our cohort did not include patients with inflammatory bowel disease, which is the most well-known disease affecting PGE-MUM. However, the possibility that other inflammatory factors may have influenced PGE-MUM levels cannot be ruled out. Third, PGE-MUM levels may be affected by several drugs. Patients who were administered laxatives were treated were excluded from our study. However, in clinical practice, patients often need to be administered laxatives and other drugs that may affect PGE-MUM during the postoperative course. In fact, most patients (84.3%) received NSAIDs during perioperative period in the present study. Although NSAIDs administration was not significantly associated with pre- and postoperative PGE-MUM levels in our cohorts, that might be because of the small number of patients who did not receive NSAIDs. Thus, further investigation that considers the effects of these drugs is needed to apply our results to clinical practice.

## CONCLUSION

The results of the present study indicate that elevated preoperative PGE-MUM levels are associated with tumour progression and that postoperative PGE-MUM levels are a promising biomarker for survival in patients with resected NSCLC. Prolonged elevation of PGE-MUM levels may aid in identifying patients who may benefit from adjuvant systemic therapy.

## Supplementary Material

ivac291_Supplementary_Data

## Data Availability

The data underlying this article will be shared on reasonable request to the corresponding author.

## ABBREVIATIONS

AIC: Akaike information criterion
BIC: Bayesian information criterion
CI: Confidence interval
COX: Cyclooxygenase
COPD: Chronic obstructive pulmonary disease
HR: Hazard ratio
MRD: Minimal residual disease
NSAIDs: Non-steroidal anti-inflammatory drugs
NSCLC: Non-small-cell lung cancer
OS: Overall survival
PGE2: Prostaglandin E2
PGE-MUM: Prostaglandin E-major urinary metabolite
